# The Quest for Improving Treatment of Cancer of Unknown Primary (CUP) Through Molecularly-Driven Treatments: A Systematic Review

**DOI:** 10.3389/fonc.2020.00533

**Published:** 2020-05-08

**Authors:** Roberta Lombardo, Federica Tosi, Annunziata Nocerino, Katia Bencardino, Valentina Gambi, Riccardo Ricotta, Francesco Spina, Salvatore Siena, Andrea Sartore-Bianchi

**Affiliations:** ^1^Niguarda Cancer Center, Grande Ospedale Metropolitano Niguarda, Milan, Italy; ^2^Department of Oncology and Hemato-Oncology, Università degli Studi di Milano, Milan, Italy

**Keywords:** cancer of unknown primary (CUP), next generation sequencing (NGS), genomic alterations, comprehensive genomic profiling, targeted therapy

## Abstract

**Background:** Carcinomas of unknown primary (CUP) account for 3–5% of all malignancy and, despite a reduction in incidence, the overall survival has not improved over the last decade. Chemotherapy regimens have not provided encouraging results. New diagnostic technologies, such as next generation sequencing (NGS), could represent a chance to identify potentially targetable genomic alterations in order to personalize treatment of CUP and provide insights into tumor biology.

**Methods:** A systematic review of studies of patients with CUP, whose tumor specimen was evaluated through a NGS panel, has been performed on June 10th, 2019 according to PRISMA criteria from PubMed, ASCO meeting library and Clinicaltrial.gov. We have identified potentially targetable alterations for which approved/off-label/in clinical trials drugs are available. Moreover, we have included case reports about CUP patients treated with targeted therapies driven by NGS results in order to explore the clinical role of NGS in this setting.

**Results:** We have evaluated 15 publications of which eleven studies (9 full-text articles and 2 abstracts) have analyzed the genomic profiling of CUPs through NGS technology, with different platforms and with different patients cohorts, ranging from 16 to 1,806 patients. Among all these studies, 85% of patients demonstrated at least one molecular alteration, the most frequent involving *TP53* (41.88%), *KRAS* (18.81%), *CDKN2A* (8.8%), and *PIK3CA* (9.3%). A mean of 47.3% of patients harbored a potentially targetable alteration for which approved/off-label/in clinical trials drugs were available. Furthermore, we have identified 4 case reports in order to evaluate the clinical relevance of a specific targeted therapy identified through NGS.

**Conclusions:** NGS may represent a tool to improve diagnosis and treatment of CUP by identifying therapeutically actionable alterations and providing insights into tumor biology.

## Introduction

Carcinomas of unknown primary (CUPs) are a heterogeneous group of metastatic tumors for which a standardized diagnostic work-up fails to identify the site of origin at the time of diagnosis (1). CUPs account for 3–5% of all malignancies (2, 3) and disappointingly the overall survival in CUP population has not improved over the last decades, despite advancements in the knowledge of biology of solid tumors (4). This is partly due to a lack of therapeutic options, with chemotherapy regimens using either platinum or taxanes or both not having proved to prolong survival in patient with CUP (5). Based on the available categorization of CUPs into favorable and unfavorable groups according to histopathological and clinical patterns (1, 6), great efforts have been done to predict the organ tissue of origin of CUPs through the IHC, DNA sequencing and gene expression analyses with the aim to better customize therapy and possibly improve clinical outcome (7–9). Based on the assumption that a treatment directed to the molecularly predicted tissue of origin could improve clinical outcome (10), a recent randomized phase II trial comparing site-specific treatment based on gene expression profiling vs. carboplatin and paclitaxel for patients with CUP has been performed. This study however demonstrated that site-specific therapy does not result in a significant survival improvement compared with empirical chemotherapy (11), leaving a clear unmet need for this patient population. Clinical outcome of CUPs is unpredictable because, even if a primary tumor does exist, they behave and metastasize unpredictably from the known primary counterpart (6) and maybe this is their real secret: their unknown biology rather than their unknown primary. CUPs enigma is hidden in the molecular mechanism that causes a fast cellular dedifferentiation and spreading (12). The aim of this review is to describe genes and molecular pathways involved in CUP pathogenesis and focus on available data of targeted genotype-directed treatment in this setting.

## Methods

### Data Sources and Search Strategy

**Figure 1:**
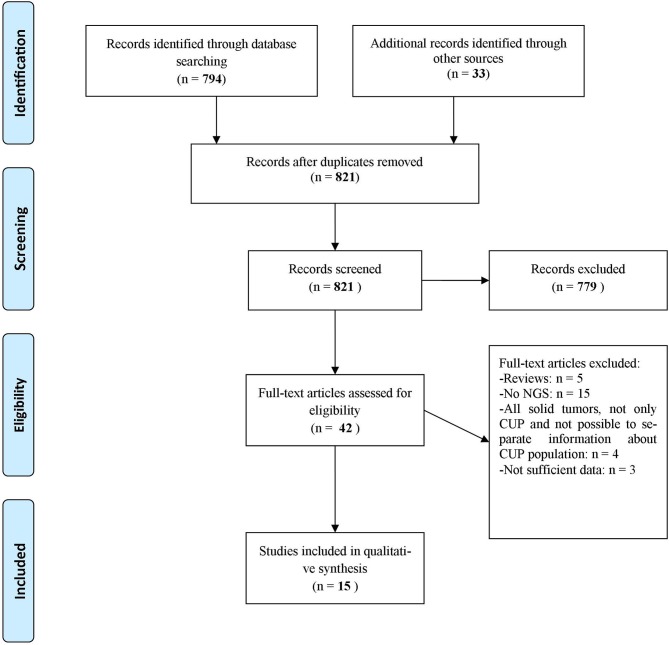
Flow diagram representing the systematic review process performed according to PRISMA Statement (13).

A systematic literature review was performed on June 10th, 2019 according to PRISMA Criteria of 2009 (13) (Figure 1). We reviewed PubMed, ASCO Meeting Library and ClinicalTrials.gov for ongoing trials. The search criteria were limited to human studies published in English language. The Medical Subject Headings terms used for the search in PubMed were [(CUP OR cancer of unknown primary) AND (NGS OR next generation sequencing OR genomic alterations OR targeted therapies)]. The Medical Subject Headings used for the search in ASCO Library were [(“CUP” OR “cancer of unknown primary”) AND (“NGS” OR “next generation sequencing” OR “genomic alterations” OR “targeted therapies”)]. The Medical Subject Headings terms used for the search in ClinicalTrials.gov were (“cancer of unknown primary site” as condition/disease).

### Selection Criteria

To be included in this review, a publication had to fulfill the following inclusion criteria: study performed in patients with cancer of unknown primary, whose tumor specimen could be evaluated through a NGS panel. We also included case reports and abstracts about CUP patients treated with targeted therapies based on the NGS results provided. The exclusion criteria were: publications written in language other than English and studies using a method of genomic analysis different from NGS.

### Article Analysis

Among the articles included in the systematic review according to selection criteria, we evaluated all the studies performed in patients with cancer of unknown primary, comparing the results obtained through different NGS panels in each study, in order to understand how many and what are the genomic alterations that could be targeted with approved/off-label/in clinical trials drugs. We also included case reports about CUP patients treated with targeted therapies based on the NGS results provided, in order to understand if NGS could represent a valid therapeutic option in real life, improving the progression free survival and the response rate of the disease.

## Results

We identified 794 records through database searching and 33 additional records through other sources (i.e., online meeting library) (Figure 1). A total of 42 records were then screened to be included in the systematic review. Twenty-seven records were excluded for the following reasons: 5 were review articles (4, 9–12), 15 studies evaluated CUP molecular profile through techniques other than NGS (7, 14–27), 4 studies took into account all solid tumors and it was not possible to separate information about CUP population from other disease conditions (28–31), 1 study evaluated the validity of NGS in establishing a clonal relationship among metastasis and with an antecedent malignancy in a CUP population (32) and 2 abstracts contained few data to allow their inclusion in the review (33, 34). As a result, 15 publications were eligible and included in the systematic review of which 9 full-text articles studies about different CUP populations and their genomic alterations (35–43), 2 abstracts about CUPs and their genomic alterations (44, 45), 1 abstract about a case report in which NGS was performed with subsequent therapeutic decisions tailored on NGS results (46), 3 articles were case reports (47–49). Other 9 case reports were extrapolated from the abovementioned full-text articles. Table 1 displays studies included in the systematic review and Table 2 shows the case reports, each with its own genomic profile obtained through NGS and the subsequent response to targeted therapy.

**Table 1:** Summary of full-text articles and abstracts of studies in CUP patients with reported genomic alterations as analyzed through different NGS panels and potentially matched drugs.

	**Tothill et al. (40)**	Gatalica et al. (43)^*^	**Ross et al. (35)**	**Löffler et al. (36)**	**Kato et al. (37)**	**Subbiah et al. (38)**	**Varghese et al. (41)**	**Clynick et al. (39)**	**Gatalica et al. (42)**	**Varghese et al. (45)**	**Chandler et al. (44)**	**Matched drugs**
N. pts	16	1806	200	55	442	17	150	17	389	34	CUP 35	
Gene/Panel	701 genes	47 genes	287 genes	50 genes	54-70 genes, ctDNA	255 genes	341-410 genes	2 gene panels, 76 genes	592 genes	341 genes	592 genes	
TP53	10	38.0%	110	30	164	5	No numbers provided. The most commonly mutated genes were TP53, KRAS, CDKN2A, KEAP1 and SMARCA4.	8	207/386	19	19	APR-246^c^
KRAS	2 (G12C)	18.0%	40	10	82	1		2	84/387	7	4	MEK-i^a^^,^^c^, KRAS G12C-i^c^
CDKN2A	3	.	37	12	8	3		2	29/356	7	3	CDK4/6-i (e.g., abemaciclib)^c^
PIK3CA	3 (2 pts E81K - VUS) (1 pt E545K)	8.0%	17	2	68	3		1	33/386	.	4	Alpelisib^a^, AKT-i^b^^,^^c^, mTOR-i^c^
AKT1	1 (E17K - VUS)	2.0%	.	3	2	.		.	.	present^*^	.	AKT-i (e.g., ipatasertib)^b^^,^^c^, mTOR-i^c^
MET	1 (R400S - VUS)	4.0%	5	2	15	.		2	.	.	.	MET TKIs (e.g., crizotinib^a^^,^^c^, tepotinib^c^)
FGFR1	.	0.0%	4	2	19	2		1	.	.	.	FGFR-i (e.g., infigratinib, ponatinib, rogaratinib)^c^
FGFR2	.	.	4	.	4	.		.	.	.	.	FGFR-i (e.g., erdafitinib^a^^,^^c^, ponatinib^c^)
FGFR3	1 (T742I - VUS)	.	.	5	3	.		.	.	.	.	FGFR-i (e.g., erdafitinib^a^^,^^c^, rogaratinib^c^)
JAK2	1 (HLG)	0.0%	.	1	1	.		.	4/351	.	.	Ruxolitinib^a^^,^^c^
CCND1	1 (HLG)	.	.	.	3	2		1	13/378	.	.	CDK4/6-i^c^
CCND2	.		4	.	2	.		.	.	.	.	CDK4/6-i^c^
BRCA2	1	11.0%	11	.	.	1		.	5/389	.	.	PARP-i (e.g., Olaparib^a^^,^^b^^,^^c^, Talazoparib^a^^,^^c^)
BRCA1	3 (1 del) (2 SNV)	0.0%	.	.	.	.		.	4/386	.	.	PARP-i (e.g., Olaparib^a^^,^^b^^,^^c^, Talazoparib^a^^,^^c^)
PTCH1	1 (S1203Afs^*^52)	.	.	.	.	.		.	5/352	.	.	Vismodegib^b^
IDH1	1 (R132L)	.	.	.	.	.		.	7/387	.	.	Ivosidenib^a^^,^^c^, olutasidenib^c^
NOTCH1	3	.	5	.	.	1		.	9/377	.	.	Bimiralisib^c^
MLH1	2	.	.	.	7	.		1	.	.	.	Immune checkpoint-i^c^
MCL1	.	.	19	.	.	1		.	9/378	.	.	Seliciclib, MIK665^c^
PTEN	.	3.0%	14	2	10	.		.	15/371	.	2	Alpelisib, AKT-i, mTOR-i^c^
ERBB2	.	2.0%	16	3	16	1		1	5/389	present^*^	.	antiHER2 (e.g., trastuzumab, pertuzumab)^a^^,^^b^
RICTOR	.	.	12	.	.	.		.	.	.	.	TORC1/2-i, mTOR-i^c^
BRAF	.	3.0%	11	3	33	.		2	16/383	present^*^	3	BRAF-i+MEK-i (e.g., vemurafenib +cobimetinib)^b^
NF1	.	.	8	.	.	.		.	7/340	.	.	mTOR-i^c^
EGFR	1	1.0%	6	3	26	.		.	0	.	.	EGFR TKIs (e.g., erlotinib^a^^,^^c^,gefitinib^*a*^)
ALK	.	.	2	1	.	1		.	.	.	.	ALK-i (e.g., alectinib^a^^,^^c^, brigatinib^a^)
RET	.	.	1	.	3	.		.	.	.	.	RET-i (e.g., pralsetinib^c^, selpercatinib^c^, lenvatinib^a^)
STK11	3	6,0%	13	.	4	.		.	18/387	.	.	mTOR-i^c^
ROS1	.	.	1	.	.	.		.	.	.	.	ROS1-i (e.g., entrectinib^a^^,^^b^)
TML-high	.	.	.	.	.	.		.	46/389	.	.	Atezolizumab^b^^,^^c^
MSI-H	.	.	.	.	.	.	.	7/389	.	.	Pembrolizumab^a^^,^^c^	
Comments	12 alterations potentially targetable: 5 with strong clinical evidence of a drug efficacy; 2 pre-clinical evidence of a drug efficacy; 5 with limited evidence	96% alterations with potential drug benefit (mainly through protein biomarkers); 98% alterations with potential drug lack of benefit	96% cases with at least one alteration; 85% cases with potentially targetable alterations: 26 with approved therapies; others with off-label therapies or in clincal trials	84% total alterations of which 15% potentially targetable	1368 total alterations of which 600 VUS. 688 alterations potentially targetable with off-label drugs; 65 alterations targetable with drugs in clinical trials	88% cases with at least one alteration. 65% cases targetable alterations with drugs in clinical trial	30% cases with potentially targetable alterations	81% cases with at least one alteration of which 52% potentially targetable with drugs approved for another indication or in clinical trials	28% cases potentially targetable by immune checkpoint-i	334 total alterations. 41% cases with potentially targetable alterations		

* *Absolute numbers not provided*;

a *FDA/EMA approved in other indications*;

b *Undergoing clinical evaluation for CUPs*;

c *Undergoing clinical evaluation for other indications*.

Eleven studies have analyzed the genomic profiling of CUPs through Next Generation Sequencing technology, with different platforms and with a different patients cohort, ranging from 16 to 1806 patients. Among all these studies (35–39), 85% of patients demonstrated at least one molecular alteration, with a mean of 47.3% of patients harboring a potentially targetable alteration for which approved/off-label/in clinical trials drugs were available. The most frequent alterations involved *TP53* (41.88%), *KRAS* (18.81%), *CDKN2A* (8.8%), and *PIK3CA* (9.3%).

In the study by Ross et al. (35), one of the biggest in this setting including 200 patients, 96% of cases harbored at least one alteration and 85% of cases showed at least one genomic alteration that could be targeted. The most common clinically relevant alterations potentially targetable included *KRAS* (20%), *CDKN2A*(19%), *MCL1* (10%), *PTEN* (7%), *PIK3CA* (9%), *ERBB2* (8%). Twenty-six alterations were associated with targeted therapies approved in a known primary tumor type; in 14 cases there were alterations targetable with off-label drugs. Furthermore, this study identified 6 cases showing activating *EGFR* mutations.

In the study by Löffler et al. (36) the most frequently mutated genes in CUP population were *TP53* (55%), *KRAS* (16%), *CDKN2A* (9%). In 15% of patients, they found alterations targetable by currently approved drugs. Collaterally, the investigators of this study observed that mutations of *KRAS* and *CDKN2A* were associated with poor PFS and females with *wild type TP53* diseases had significantly better PFS and OS in comparison with male population.

In order to overcome both genomic heterogeneity between the primary tumor and all the metastatic lesions and temporal molecular changes occurring during sequential therapies, Kato et al. (37) analyzed the genomic profile of a CUP population of 442 patients using NGS applied on circulating tumor DNA (ctDNA). They found at least a genomic alteration in the 80% of cases, the most common of which interesting *TP53* (37%), *KRAS* (18%) and *PIK3CA* (15%). Though, approximately 44% of the abovementioned alterations were variants of unknown significance (VUS). 50% of 1368 alterations were potentially targetable with off-label/in clinical trial drugs, whereas 63.8% of patients showed an alteration targetable with an FDA-approved agent. With this retrospective study Kato et al. demonstrated how the tumor molecular evolution during several lines of therapies could be pursued using NGS on ctDNA, with the possibility to customize the therapy time by time, also avoiding invasive biopsies.

In the study by Gatalica et al. (43) the most commonly mutated genes were *TP53* and *KRA*S (38 and 18%, respectively), followed by *BRCA2, PIK3CA*, and *STK11* (with a frequency ≥5%); the most commonly amplified genes were *EGFR* and *HER2* (17 and 5%, respectively).

In other three studies by Tothill et al. (40), Subbiah et al. (38), and Clynick et al. (39), 75, 65, and 52%, potentially targetable alterations with approved/approved for another indication/in clinical trials drugs were found, respectively. The most common clinically relevant alterations detected in these studies included *ERBB2, EGFR, KRAS, PIK3CA*, and *BRAF*. Clynick et al. used two different gene panels, each one detecting a different number of alterations, 8 alterations were detected by both panels. Subbiah et al. also evaluated the clinical response in 7/17 patients who received a therapy matched to molecular aberrations (Table 2): the best tumor responses were stable disease lasting up to 6 months in 3 patients and 1 remaining on therapy for over 8 months.

In the study by Varghese et al. (41), including 150 patients, 54 potentially targetable alterations were identified in 45 patients. The most commonly mutated genes were *TP53, KRAS, CDKN2A, KEAP1*, and *SMARCA4*. Twenty-seven targetable alterations with FDA-approved drugs for another indication were found in 23 tumors (most common *ERBB2* amplification and *BRAF V600E* mutation); 27 alterations targetable with drugs for which a clinical evidence exists but in another indication were found in 25 tumors (most common *PIK3CA* mutation); 32 alterations targetable with drugs for which preclinical evidence exists were found in 38 tumors (the most common *KRAS* mutation). Fifteen patients in the study received a targeted therapy shown to be active in patients with *BRAF V600E* mutations, *ERBB2* amplification, *KIF5B-ALK* fusion and *NCOA4-RET* fusion. Among them, results in terms of time to treatment failure (TTF) were variable, ranging from <1 month to 14 months, and several patients remaining on therapy at the time of data cut-off.

Gatalica et al. (42) studied a CUP population of 389 patients in which tumor mutational load (TML) and microsatellite instability (MSI) were evaluated through NGS, while *PD-L1* expression using immunohistochemistry (with 5% cut-off value). High TML was detected in approximately 12% of patients, MSI-high (MSI-H) in 2% of patients, expression of *PD-L1* >5% in 22% of cases. Furthermore, predictive biomarkers of hyperprogression to immune checkpoint inhibitors, including *MDM2* gene amplification and loss-of-function *JAK2* gene mutations, were identified in 2 and 1% of cases, respectively.

Finally, in the study by Chandler et al. (44), in a population of different solid tumors of which 1,172 samples could be analyzed with NGS, CUPs represented the 3%, and among these, the most frequently alterations interested *TP53* (54%), *KRAS* (11%), *PIK3CA* (11%), *BRAF* (9%). Also in the study by Varghese et al. (45), in a population of 34 patients, 334 alterations were identified, most commonly in *TP53* (19/34), *CDKN2A* (7/34), *KRAS* (7/34); potentially targetable alterations were identified in 14/34 of patients and included *BRAF V600E, ERBB2 S310F*, and *AKT1 E17K*.

In the absence of prospective clinical trials, the clinical relevance of a specific targeted therapy identified through NGS, is coming from case reports. Table 2 displays case reports retrieved in this systematic review.

## Discussion

Results of this systematic review from nine published studies and two abstracts show that 85% of patients with CUP harbor in their tumor at least one identified genomic alteration, including variants of uncertain significance (VUS), and that 47.3% of them present a potentially targetable alteration for which approved/off-label/in clinical trials drugs are available. The most frequent alterations were found in *TP53, KRAS, CDKN2A, PIK3CA*; interestingly none of the patients had two identical molecular profiles underlying the assumption that CUPs are an individually heterogeneous molecular and clinical entity.

Although there are no targeted therapies, *TP53* is one of the most frequently altered gene in CUPs. This molecular alteration appears to be associated with high *VEGF-A* levels (50) and clinical data suggest that patients with *TP53* mutations have better progression-free survival (51) and improved clinical outcome with anti-VEGF drugs (52) in comparison with patients with *wild-type TP5*3. *RAS*-driven tumors are potentially targetable with *MEK* inhibitors (e.g., trametinib and cobimetinib) (53) and some ongoing trials are evaluating the activity of different drugs against *KRAS G12C* mutation (54, 55), while *PIK3CA* mutations have been shown to be associated with response in 45% in patients with advanced cancers when treated with a *PI3K* inhibitor or *mTOR* inhibitors (56); further, the specific inhibitor alpelisib has recently gained FDA-approval for breast cancer (57). Until now, measuring the clinical value of the genomic alterations identified in the CUP population has been elusive, because only case reports are available about targeted therapies customized according to individual genomic profiles (Table 2). The ongoing clinical trials will better elucidate the clinical validity of this approach for CUPs (Table 3).

**Table 2:** Case reports of CUP patients with the genomic profile obtained through NGS panels and clinical outcome to matched targeted therapy.

**References**	**Histology and mutational profile provided by NGS**	**Therapy**	**Results**
Ross et al. (35)	Histology not specified, EML4-ALK fusion	Crizotinib	Best response: PR
Kato et al. (37)	Adenocarcinoma of unknown primary (ACUP), KRAS G12D, MLH1 R389W mutation	Trametinib+Nivolumab	Best response: PR
Subbiah et al. (38)	Squamous cell carcinoma of unknown primary, PIK3CA H1047R, KDM6A S466, ALK L560F, CDC73 Q333, SOX2 amplification, CDK12 Q570	PI3K-i	Best response: SD. PFS = 6.5 months
	ACUP, FBXW7 splice 726+1 G>A, APIP-NOTCH1 fusion, FGFR1 amplification, TP53 L45P, Q38fs79, ARID1A Y1211fs5, MYST3 amplification, ETV1 rearrangement	Carboplatin+bevacizumab+temsirolimus	Best response: SD. PFS > 8 months
	Carcinoma of unknown primary (CUP), FBXW7 R465H, CCNE1 amplification, PIK3CA Q75E, TP53 R273C	Everolimus+anastrozole	PD
	CUP, FBXW7 W244, TP53 R248Q	Sirolimus+hydroxychloroquine	Best respone: SD. PFS = 4.5 months
	CUP, PIK3CA E545K and FGFR1 amplification, SOX2 amplification, KRAS amplification, TP53 R196, CCND1 amplification, CDKN2A/B loss	Lenalidomide+temsirolimus	PD
	Squamous cell CUP, BRCA2 W1692fs3, ARID1A S1929fs25, CDKN2A/B loss, EMSY amplification, MDM2 amplification, SMAD4 P130S, SOX2 amplification	Liposomial doxorubicin+bevacizumab +temsirolimus	PD
	CUP, NF2 splice site 448-1G>A	Lapatinib+sirolimus	PD
Tan et al. (48)	Poorly differentiated carcinoma, EGFR L858R	Gefitinib	Best response: PR. PFS = 11 months
Palma et al. (47)	Poorly differentiated carcinoma, EGFR WT, KRAS G12V mutation, MET amplification, CCND1/MYC/TP53/CARD11 amplification	Crizotinib	Best response: CR. PFS = 19 months
Gröschel et al. (49)	Poorly differentiated adenocarcinoma, TP53 E135fs, KRAS G12S, mutations of uncertain significance in PI3KCD, CDKN2A,NCOA1, FAT2, EGFR,ARID1A, overexpression of PDL1	Pembrolizumab	Best response: Near CR
Zhao et al. (46)	CUP, EML4-ALK fusion	Crizotinib	Best response: PR

**Table 3:** Recruiting studies available for CUP population.

**Study (study ID) Phase Main location**	**NGS**	**Drugs**
A Phase II Randomized Study Comparing the Efficacy and Safety of Targeted Therapy or Cancer Immunotherapy vs. Platinum-Based Chemotherapy in Patients With Cancer of Unknown Primary Site (CUPISCO) Phase II 116 study locations	Yes	Alectinib Vismodegib Ipatasertib Olaparib Erlotinib Bevacizumab Vemurafenib Cobimetinib Trastuzumab Pertuzumab Atezolizumab Carboplatin Paclitaxel Cisplatin Gemcitabine
Trial of Pembrolizumab in Cancer of Unknown Primary (CUPem) Phase II London, United Kingdom	No	Pembrolizumab
Tissue-of-origin Directing Therapy in Patients With Cancer of Unknown Primary Phase III Shangai, China	No	Standard treatments based on tissue-of-origin (ORIGIN-PanCA°R) profiling vs. standard empiric chemotherapy
Ontario-wide Cancer TArgeted Nucleic Acid Evaluation (OCTANE) Observational Ontario, Canada	Yes	__
Genomic Investigation of Unusual Responders (GENIUS) Observational Ontario, Canada	Yes	__
Pembrolizumab in Patients With Poor-Prognosis Carcinoma of Unknown Primary Site (CUP) (CUP) Phase II Alberta, Canada	No	Pembrolizumab

Potential limitations of a tissue-agnostic therapeutic approach include that extrapolating therapeutic actionability from one cancer histology to another might provide uncertain results: for example, known differences exist in the clinical activity of *BRAF* inhibitors in melanoma and colorectal cancer (58, 59) and in the efficacy of *HER2*-directed treatments in breast as compared to gastric (60, 61) or colorectal cancers (62). Therefore, for CUP patients it would be still important to consider putative primary sites even when candidate actionable driver mutations are found. Finally, we should take into account that redundancy in activation of pathways of resistance does often take place as a mechanism of primary as well as secondary resistance (63, 64), for example through the co-activation of two or more antagonist pathways, affecting opportunities for a targeted pharmacological blockade (29). Liquid biopsies have a high sensitivity and specificity in reflecting the onset of resistance mutations as well as in detecting tumors alterations (65) and they could be used for these goals. Kato et al. (37) observed that NGS on blood-based biopsies can reveal clinically relevant genomic alterations in CUPs, occurring during different lines of therapy, enabling precision medicine, and liquid biopsy may also reflect the biological heterogeneity of the tumor in time and between the primary and the metastatic sites, providing more insights in CUPs biology and natural history.

Based on the assumption that, as far as they can evolve, metastatic lesions should retain the signature of the primary tumor (66), several trials have been performed using gene expression profiling techniques such as RT-PCR assay (10, 67) or using epigenetics by identifying the DNA methylation profile of the primary (15, 68), in order to identify the potential tissue of origin and drive therapy accordingly. However, based on the recent study by Hayashi et al. (11) it is still questionable whether a site-specific chemotherapy is beneficial and further studies are needed to define this point.

Multiple oncogenic pathways have been investigated in order to understand their contribution in pathogenesis of CUPs, their prognostic value and value as therapeutic targets. With this regard, we found that the most frequent alterations in CUPs involve *TP53, KRAS, CDKN2A, PIK3CA*. Pentheroudakis et al. (69) and Kampsorias et al. (70) did not find a prognostic value by analyzing the expression of *TP53, RAS, C-Myc*, and *Bcl-2*, as well as *EGFR* or *HER2* overexpression; furthermore, they noticed that the incidence of tumor suppressor and DNA repair gene inactivation in CUPs is similar to that reported in other solid tumors with a known primary site. According to Löffler et al. (36), mutations of *KRAS* and *CDKN2A* are associated with poor PFS, while *wild type TP53* in females has a positive prognostic value associated to a significantly better PFS and OS in comparison with male population. Karvasilis et al. (18) also studied the tissue expression of *VEGF* and *TSP-1* in CUPs, the first with a role of activator of angiogenesis and the second as an endogenous inhibitor of the process and they found a negative association between *TSP-1* expression and microvessel density, suggesting that *TSP-1* can correlate with a favorable prognosis, furthermore, microvessel density was low in the group of favorable CUPs, but none of the abovementioned factors showed a prognostic value. Stella et al. (71) evaluated the role of *MET* in a heterogeneous population with a known or unknown primary. *MET* activation is a late event in tumorigenesis, after the onset of unfavorable microenvironmental conditions, and it was found to be mutated in all CUPs samples, associated with a negative prognosis; in our review we found that *MET* alterations are present in 2.7% of cases (35–37, 39, 40, 43) and, as demonstrated in a case report (35, 47), the targeted agent Crizotinib is associated with a better outcome in this setting. Gatalica et al. (43) noticed that the overexpression of two topoisomerases (Topo1 and Topo2alfa), identified through immunohistochemistry analysis, was associated with a potential benefit using cytotoxic therapies; on the contrary, the overexpression of multidrug resistance–associated protein 1 (MRP1) and the overexpression of breast cancer resistance protein (BCRP), a member of the ABC transporter proteins, were associated to a potential drug lack of benefit. Also the microenvironment could play a role in determining a dormancy of the primary tumor cells (72), as well as metalloproteinases allowing tumor cells migration (70) and Programmed Death-1 (PD-1) T cell co-receptor and its ligands, B7-H1/PD-L1 and B7-DC/PD-L2, maintaining an immunosuppressive tumor microenvironment (73). Gatalica et al. (42, 43) found that *PD-L1* expression, detected through immunohistochemistry (with 5% cut-off value), was present in 22% of cases; while a high TML, detected through NGS, was expressed in 12% of patients and MSI-H status in 2% of patients. Ongoing clinical trials are evaluating the clinical impact of immune checkpoint inhibitors in CUP population (Table 3).

Altogether these data highlight the absence of an established distinguishing underlying molecular biology make-up of CUPs, confirming that their molecular pathogenesis is complex and heterogeneous. In order to overcome this lack of knowledge, NGS represents a chance, although not validated by clinical trials, to improve diagnosis and matched treatment of potential actionable molecular alterations.

## Author Contributions

RL: writing of the article, analysis, and interpretation of data. AN, KB, VG, RR, FS, and FT: writing of the article and critical revision of the draft. SS: conception and design of the review, analysis and interpretation of data, critical revision of the draft, and final approval of the version to be submitted. AS-B: conception and design of the review, writing of the article, analysis and interpretation of data, critical revision of the draft, and final approval of the version to be submitted.

## Conflict of Interest

AS-B has acted as a consultant/advisory member for Amgen, Bayer, Lilly, and Merck- Serono. SS is advisory board member for Amgen, Bayer, BMS, Celgene, Incyte, Merck, Novartis, Roche, Seattle Genetics. RR is consultant/advisory member for Novartis, Pfizer, Janssen-Cilag, MSD, BMS, Sandoz, Sanofi Genzyme. The remaining authors declare that the research was conducted in the absence of any commercial or financial relationships that could be construed as a potential conflict of interest.
